# Transgenic Plants as Low-Cost Platform for Chemotherapeutic Drugs Screening

**DOI:** 10.3390/ijms16012174

**Published:** 2015-01-20

**Authors:** Daniele Vergara, Stefania de Domenico, Michele Maffia, Gabriella Piro, Gian-Pietro Di Sansebastiano

**Affiliations:** 1 Department of Biological and Environmental Sciences and Technologies (DiSTeBA), University of Salento, Campus ECOTEKNE, S.P. 6, Lecce-Monteroni, Lecce 73100, Italy; E-Mails: michele.maffia@unisalento.it (M.M.); gabriella.piro@unisalento.it (G.P.); gp.disansebastiano@unisalento.it (G.-P.D.S.); 2 Laboratory of Clinical Proteomics, Vito Fazzi Hospital, Lecce 73100, Italy; 3 Institute of Science of Food Production, National Research Council (CNR), Lecce 73100, Italy; E-Mail: stefaniadedomenico@yahoo.it

**Keywords:** *Arabidopsis thaliana*, chemotherapeutics, cytoskeleton, endomembrane trafficking, green fluorescent protein

## Abstract

In this work we explored the possibility of using genetically modified *Arabidopsis thaliana* plants as a rapid and low-cost screening tool for evaluating human anticancer drugs action and efficacy. Here, four different inhibitors with a validated anticancer effect in humans and distinct mechanism of action were screened in the plant model for their ability to interfere with the cytoskeletal and endomembrane networks. We used plants expressing a green fluorescent protein (GFP) tagged microtubule-protein (TUA6-GFP), and three soluble GFPs differently sorted to reside in the endoplasmic reticulum (GFPKDEL) or to accumulate in the vacuole through a COPII dependent (AleuGFP) or independent (GFPChi) mechanism. Our results demonstrated that drugs tested alone or in combination differentially influenced the monitored cellular processes including cytoskeletal organization and endomembrane trafficking. In conclusion, we demonstrated that *A. thaliana* plants are sensitive to the action of human chemotherapeutics and can be used for preliminary screening of drugs efficacy. The cost-effective subcellular imaging in plant cell may contribute to better clarify drugs subcellular targets and their anticancer effects.

## 1. Introduction

The search for more effective human therapies is one of the most important objectives of modern chemistry and biology. The introduction of cancer therapeutic agents that target specific molecular pathways is now a hallmark in oncology and the basis of a future personalized medicine. The development of a new drug has become now increasingly dependent on an understanding of tumor biology and the path to success for targeted-molecules, strongly correlated to the comprehension of their biological mechanism of action including possible side effects. Genomic studies described numerous genomic alterations including human epidermal growth factor receptor-2 (Her2-neu), anaplastic lymphoma kinase (ALK), KRAS, and Bcr/Abl, against which a large library of small molecules have been tested. Together with the discovery of new tumor mutations, new molecules are continuously synthesized and efficient screening methods for their functional characterization are thus required [1]. A limiting factor is the cost of screening studies to identify the subcellular and molecular targets of new compounds. To date, screens for chemotherapeutics have been largely performed *in vitro* using either cultured cell lines, or cells isolated from freshly dissected tissues [2]. Tumor cells can also be implanted *in vivo* using appropriate animal models to investigate the action of a specific drug on tumor microenvironment and to integrate pharmacokinetic and pharmacodynamic investigations. Efforts are also underway to characterize drug action against potential off-targets. In fact, besides the active inhibition of a specific ligand, several chemotherapeutics can potentiate their cytotoxic effects through the modulation or other pathways and the interplay with several cellular specialization [3]. This can have several implications for patient outcome including drug-side effects.

More recently, biomedical research has pushed forward the exploration of new models including zebrafish (*Danio rerio*) and *Drosophila* as a high-throughput and cost-effective alternatives to current animal models for the rapid screening of several compounds [4,5]. Higher plants also represent a suitable system for the risk assessment of chemicals and formulations of human relevance [6]. For example, it has been shown that *A. thaliana* transgenic lines are applicable for small molecules and drugs screening [7], but even more rapid and effective approaches may be designed for specific and limited purposes.

Here, we propose to use model plants to characterize conventional anticancer drugs effects on eukaryote subcellular targets, the cytoskeleton and the endomembrane system, both often involved in cellular processes leading to different human diseases. In particular, this work aims to test the correspondence and significance of the observed alteration in plant cells with the effects expected from known chemotherapeutic drugs. Alterations in the cellular cytoskeletal architecture characterize tumor samples with different metastatic potential and cytoskeletal alterations are frequently observed after drug treatment [8,9,10]. The endoplasmic reticulum (ER) stress is related to several human diseases, including diabetes, neurodegeneration, and cancer [11], and associated to drug action or drug side effects [12,13]. Moreover, a comparison of the annotated *A. thaliana* and human genome sequences reveals that a high percentage of genes implicated in cytoskeletal organization and vesicle trafficking are also present in *A. thaliana* [14]. Unlike mammals, the principal components of the plant cytoskeleton are microtubules (MTs) and actin filaments (AFs); intermediate filaments (IFs) have not been described in plants. Moreover, the *A. thaliana* proteome appears to lack homologues of proteins that, in animal cells, link the actin cytoskeleton to the extracellular matrix. However, in both plants and mammals, the dynamic stability of MTs and AFs is influenced by MT-severing ATPases, AF-crosslinking/bundling proteins, and AF-disassembling proteins, such as profilin and actin-depolymerizing factor/cofilin [15]. Interestingly, this genetic and functional similarity has also made possible the development of complementary approaches in which mammalian cells were used as expression system model to identify new plant cytoskeleton binding proteins [16]. Plants can then be used to screen small molecules designed to modulate the expression of this protein family.

For our screening, we selected four different classes of drugs, Taxol, Y-27632, Crizotinib, and Sorafenib, with a different mechanism of action. Paclitaxel (Taxol) is a drug of natural origin isolated from the bark of *Taxus brevifolia* (the Western yew tree) that promotes the polymerization of tubulin by blocking the disassembly of microtubules [17]. Y-27632 is an inhibitor that has as main target the activity of the Rho-associated protein kinase (ROCK), which participates in cell morphology and motility through the regulation of cytoskeletal dynamism [18,19]. Crizotinib (PF-02341066) is an ATP-competitive, small-molecule inhibitor of the receptor tyrosine kinases (RTKs) c-Met and ALK [20]. Sorafenib (BAY43-9006) is a multi-kinase inhibitor that inhibits C-Rapidly Accelerated Fibrosarcoma (RAF), B-RAF, vascular endothelial growth factor-2 and -3 (VEGFR-2, VEGFR3), platelet derived growth factor receptor-β (PDGFRβ), FMS-like tyrosine kinase 3 (Flt3), and C-Kit [21]. To visualize the drug’s effects, the cytoskeleton and the endomembrane system was visualized in *A. thaliana* plants expressing GFP-tagged protein. The characterization of bioactive chemicals effect on subcellular targets was based on *in vivo* fluorescence microscopy and confocal microscopy. The simple visual screening of drugs effect on plant tissues expressing specific fluorescent markers was also used to test drugs combinations to enhance toxic effects in the aim to optimize chemotherapy “drugs cocktails”, to be used in the treatment of cancer diseases. Subcellular imaging in transgenic *A. thaliana* plants proved to be a potentially cost-effective preliminary screening tool to evaluate human chemotherapeutics and eventually to further investigate drugs anticancer effects.

## 2. Results

**Figure 1:**
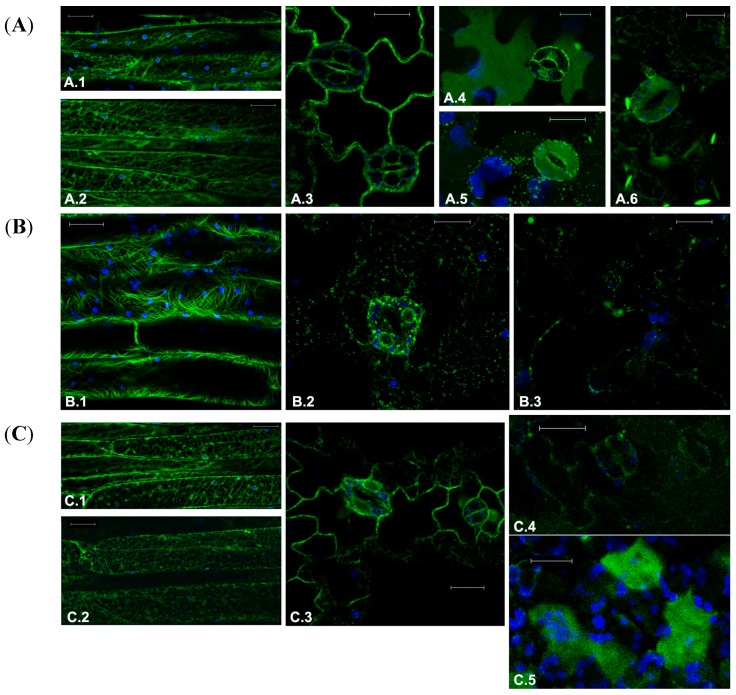
Fluorescent patterns of GFP-tagged marker proteins in transgenic Arabidopsis tissues in control conditions or treated with drugs targeting the cytoskeleton. (**A**) Normal fluorescent pattern of transgenes of (**A.1**) and (**A.2**) microtubules marker GFP-TUA6 in elongated petiole cells; (**A.3**) ER marker GFPKDEL in leaf epidermal cells; (**A.4**) Marker of the lytic vacuole, AleuGFP, in the lower face of the leaf; (**A.5**) AleuGFP, in the upper face of the leaf; and (**A.6**) Vacuolar marker characteristic of the direct transport ER-to-vacuole GFPChi; (**B**) Effect of treatment with 30 μM Paclitaxel on fluorescent pattern of (**B.1**) GFP-TUA6 in petiole cells; (**B.2**) GFPKDEL in leaf epidermis; (**B.3**) GFPChi in leaf epidermis; and (**C**) Effect of treatment with 200 μM Y-27632 on fluorescent pattern of (**C.1**) GFP-TUA6 in petiole cells showing no significant alteration; (**C.2**) GFP-TUA6 showing stronger effects; (**C.3**) GFPKDEL in leaf epidermis; (**C.4**) GFPChi faint fluorescence in leaf epidermis; and (**C.5**) GFPChi fluorescence secreted in the intercellular spaces of leaf mesophyll. GFP fluorescence and chlorophyll autofluorescence are shown in green and blue, respectively. Scale bar = 20 μm.

The action of four different drug inhibitors was investigated in the model plant *A. thaliana*. The organization pattern of subcellular targets was made more evident by the expression of exogenous genes coding GFP-based fluorescent chimerical proteins: Cell cytoskeleton was labelled by the GFP tagged microtubule-protein TUA6 (GFP-TUA6, Figure 1A.1,A.2) [22], the endomembrane system was labeled by three soluble GFPs differently sorted to reside in the ER (GFPKDEL; Figure 1A.3) [23], to accumulate in the vacuole through a COPII dependent transport mechanism (AleuGFP; Figure 1A.4,A.5) or through a COPII independent mechanism (GFPChi; Figure 1A.6). The distribution of these two vacuolar markers is tissue specific and also depends on the activity of light regulated proteases [24]. Both markers can reach the central vacuole but they use different sorting machineries and intermediate compartments so that they reach the central vacuole with different efficiency. AleuGFP is generally visible in the central vacuole but it is more evident in epidermal cells of the epidermis of the lower face of the leaf/cotyledon, while stomata guard cells show no fluorescence in the central vacuole (Figure 1A.4); on the upper face of the leaf/cotyledon the situation is inverted with a stronger fluorescence in the vacuole of guard cells (Figure 1A.5). The detailed pattern of these markers was previously described [23].

### 2.1. Effect of Chemotherapeutic Molecules on Plant Cells

The first drug to assess the feasibility of our approach was Paclitaxel, which has been traditionally used to promote and stabilize tubulin polymerization in plants [25]. After 24 h of treatment, the dose of 10 μM induced MTs disorganization only in few cells, a result that was more effective with a higher concentration of drug. At 30 μM, Paclitaxel induced an extensive disorganization of the MTs in different cell types, and a more dramatic morphological alterations due to detachments from organelles such as plastids and the acquisition of a curvilinear appearance, probably due to excessive growth, not counterbalanced by mechanisms of depolymerization (Figure 1B.1). The dose of 30 μM was also able to affect the ER completely disorganizing its reticular structure (Figure 1B.2). With the full disorganization of the ER, also vacuolar transport was impaired so that labeling due to the vacuolar markers AleuGFP (not shown) and GFPChi nearly disappeared (Figure 1B.3).

Y-27632 moderately affected markers distribution in plant cells. The observed effects were different compared to those obtained with Paclitaxel. The doses of 25, 50, 75 and 200 μM were tested on transgenic plants inducing moderate alterations only at the higher doses. After 24 h of treatment (200 μM), some elongated petiole cells showed disturbed MTs (Figure 1C.1) but, at the same time, other cells in the same petiole had MTs with a nearly normal fluorescent pattern (Figure 1C.2). The effect on the ER was moderate too. Stomata guard cells appeared to suffer in their ability to maintain turgor but the effect on ER gave no rise to clear morphological alterations (Figure 1C.3). GFPChi distribution suffered the strongest effect. In fact, fluorescence of the reporter protein decreased in all cell types, especially in elongated cells (not shown); in the epidermis a faint fluorescence was still detectable in the central vacuole of some cells (Figure 1C.4) but in the spongy mesophyll below the epidermis, fluorescence accumulated irregularly in the intercellular spaces (Figure 1C.5). AleuGFP accumulation was also altered in a similar way but the alteration of the ER has a predictable and indirect effect on post-Golgi vacuolar transport. The alteration of AleuGFP pattern was expected and considered not informative (not shown).

**Figure 2:**
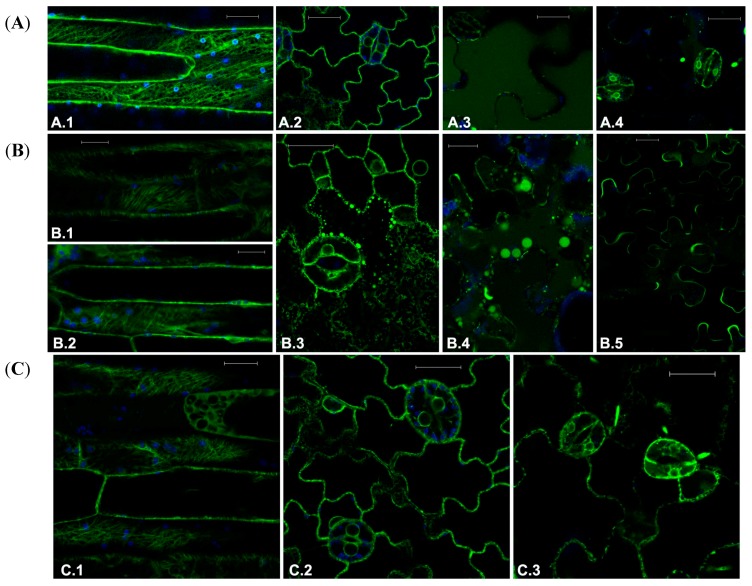
(**A**) Fluorescent patterns of GFP-tagged marker proteins in transgenic Arabidopsis tissues in control conditions (**A.1**) GFP-TUA6 in petiole cells, (**A.2**) GFPKDEL, (**A.3**) AleuGFP, and (**A.4**) GFPChi in leaf epidermis or treated with drugs targeting the endomembranes; (**B**) Effect of treatment with Crizotinib at the concentration of 0.2 μM (**B.1**) or 20 μM (**B.2**) on fluorescent pattern of GFP-TUA6 in petiole cells; (**B3**) effect of 20 μM in leaf epidermis on GFPKDEL; (**B.4**) AleuGFP and (**B.5**) GFPChi distribution; and (**C**) Effect of treatment with Sorafenib at the concentration of (**C.1**) 2 μM on GFP-TUA6 in petiole cells or 1 μM on GFPKDEL (**C.2**) and AleuGFP (**C.3**) in leaf epidermis. GFP fluorescence and chlorophyll autofluorescence are shown in green and blue, respectively. Scale bar = 20 μm.

Crizotinib is an ATP-competitive inhibitor with multiple targets and its effect was tested on transgenic plants fluorescent typical patterns (Figure 2A.1–A.4) at the concentration of 0.2 and 20 μM. The drug’s effect on MT distribution was mild but visible already at 0.2 μM (Figure 2B.1). The highest dose induced diffusion of fluorescence as due to depolymerization but MT structure was still visible (Figure 2B.2). The highest dose also induced disruption of ER organization (Figure 2B.3) and vacuolar complex alteration revealed by AleuGFP (Figure 2B.4). Interestingly this marker was sorted to the vacuole but its distribution was aberrant, showing fluorescent inclusion in the vacuole lumen. The other vacuolar marker GFPChi was mis-sorted to the apoplast even after the treatment with the lower dose of Crizotinib (0.2 μM) (Figure 2B.5). These effects suggest that the drug has high specificity for specific steps of vacuolar transport.

The multi-kinase inhibitor Sorafenib was tested at the concentration of 0.01–1 and 2 μM. The effect on MTs become visible at 1 μM when large areas of the elongated cells in the petiole (or in the hypocotyl) showed disorganized GFP florescence, but it did not increase significantly at 2 μM (Figure 2C.1). The observation suggested that only actively elongating cells showed the effect of the drug considering that MTs in the epidermis did not suffer alterations.

ER was not significantly affected (Figure 2C.2) but the vacuolar marker AleuGFP seemed to be blocked in the ER and could not be exported when plantlets were treated with 1 μM (Figure 2C.3). Being GFPChi largely accumulated in ER, its pattern was not considered informative (not shown).

### 2.2. Screening of Combination Chemotherapy on Plant Cells

Several rationally chosen drug combinations are under clinical evaluation to increase the therapeutic potential of targeted agents and to reduce potential side effects [26]. The preclinical molecular investigation of these combinations has a central role in the possible selection of anticancer drugs. Once more the availability of a cost-effective eukaryotic model to screen for combination efficacy may help focusing of preclinical molecular investigations.

**Figure 3:**
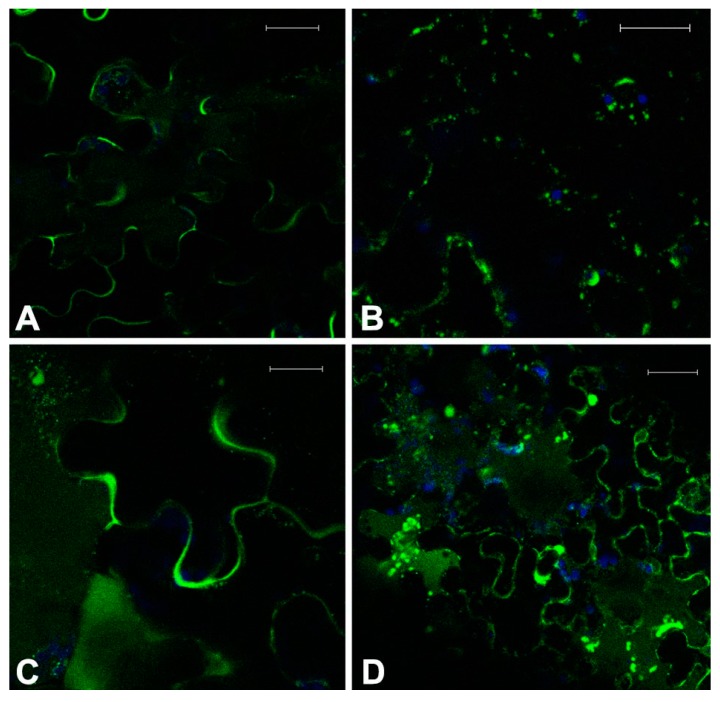
Fluorescent patterns of GFPChi in transgenic Arabidopsis leaf epidermis treated with low doses of two combined drugs. Treatment with 0.2 μM Crizotinib had a moderate effect on GFPChi (**A**); but it was enhanced when combined with 10 μM Paclitaxel disturbing intermediate steps of its sorting (**B**) or causing mis-sorting to the apoplast when combined with 25 μM Y-27632 (**C**); combined treatment with 0.01 μM Sorafenib caused the increase of fluorescence in the central and small vacuoles (**D**). GFP fluorescence and chlorophyll autofluorescence are shown in green and blue, respectively. Scale bar = 20 μm.

Plantlets were treated with a low dose of Crizotinib (0.2 μM) in combination with Paclitaxel (10 μM), Y-27632 (25 μM), and Sorafenib (0.01 μM). It was hypothesized that the observed effect was influenced by a higher stress of tissue and for this reason the alterations taken into account had to be homogeneously visible throughout the tissue examined. With a longer treatment, up to 36–40 h, Crizotinib alone (0.2 μM) was already able to induce some of the symptoms typical of higher doses. Some cell kinds, possibly not fully differentiated (e.g., cells flanking the differentiating stomata) and not used for the screening, showed to be particularly sensitive (not shown). To evaluate synergistic effects, only stronger and extensive effects were taken into consideration. In doing so, some interesting interactions were evidenced. These effects were particularly evident on GFPChi because this marker sorting was already weakly affected by 0.2 μM Crizotinib (Figure 3A).

The combination of Crizotinib and Paclitaxel caused an increased disturbance to MTs organization and, probably as a consequence, of GFPChi vacuole sorting (Figure 3B). The marker was no more able to exit the ER and the network itself appeared disorganized. The fluorescent patterns were anyhow comparable with the effect due to higher doses of the single drugs.

The combination of Crizotinib and Y-27632 appeared synergistic altering all markers. MTs and ER organization showed no significant differences from the effect of single drugs at slightly higher doses (not shown) but the effect on sorting of GFPChi marker appeared significantly stronger (Figure 3C).

The combination of Crizotinib and Sorafenib moderately increased the effect on cells MTs organization. The effect was more generalized than the effect observed with Crizotinib alone but was not significantly different in pattern and intensity (not shown). The effect on the vacuolar marker GFPChi was more interesting, in fact combination of the two drugs caused the increase of fluorescence in the central vacuoles and in small vacuoles (Figure 3D) similar to those induced by Crizotinib when AleuGFP was expressed (Figure 2B.4) and completely dissimilar from the mis-sorting of GFPChi to the apoplast normally caused by Crizotinib (Figure 2B.5).

## 3. Discussion

The interest in using transgenic plants as tools to investigate human disease mechanisms has grown in recent years [14,15,16,17,18,19,20,21,22,23,24,25,26,27]. The observation that plants have a set of cellular processes and biological functions very similar to those observed in humans have important practical implications, suggesting that it may be possible to predict the action of human potential drugs via the large screening of transgenic plants. Moreover, the fast and efficient generation of a large number of transgenic plants facilitates and supports this approach [14]. The screening method described here focused on some MTs, ER, and vacuole markers, but this method should be applicable to the study of other cytoskeletal components or the study of sorting of other known markers such as for endocytosis, unfolded protein response or degradation.

To demonstrate this potential, the first drug used to assess the feasibility of our approach was Paclitaxel (also known as Taxol). This cytotoxic compound has a well-known mechanism of action; it promotes microtubule assembly and stability, leading to the inhibition of the dynamic reorganization of the microtubular structure. Microtubules are long, filamentous, tube-shaped cytoskeletal proteins, formed by the association of α/β-tubulin heterodimers into polymers. Cytoskeleton composition is conserved among Eukaryotes and differences are restricted only to some organization aspects and specialized functions [28,29]. Consistent with these expectations, plant microtubules were sensitive to the action of Taxol that acts by inducing MTs disorganization after 24 h of treatment at a concentration that is similar to that used in human cancer models [30]. The practical implication is that plants can then be used as a reliable screening tool for understanding the mechanisms of action of novel microtubule-targeted compounds with a greater antitumor activity.

Another inhibitor that potentially targets the organization of cytoskeleton is Y-27632. Effects of Y-27632 were different compared to those obtained with Paclitaxel. In mammalian cells this drug is an inhibitor of ROCK, a key regulator of actin cytoskeleton and cell polarity. Many related upstream regulators and downstream effectors were identified including the Rho family of GTPases (Rho GTPases) and Cofilin, respectively. As stated before, actin-binding proteins (ABPs) are essential in almost every eukaryotic cell type and multiple cofilin genes were identified in *A. thaliana* [31]. Although a homolog of ROCK has not been identified in plants, there are interesting parallels in the regulation of mammalian and plant ABPs. Plants possess a single subfamily of Rho GTPases, called ROPs, with a role in cellular processes similar to those of their human counterparts, including signaling to the cytoskeleton and vesicular trafficking [32,33]. Furthermore, a wide variety of conserved ROP effector proteins are also known [33].

In plants, Y-27632 caused little effect on cytoskeleton and ER but influenced the sorting of the vacuolar marker GFPChi. This marker can bypass the Golgi being sorted directly from the ER to the vacuole and its mis-sorting to the apoplast may indicate a possible effect on endosomes recycling, leading to unconventional secretion [34]. Similarly, Rho GTPases has also been implicated in the regulation of endocytic trafficking in human cells [35] and treatment with Y-27632 can interfere with this process [36].

Crizotinib and Sorafenib are two small molecules whose clinical utility has already been demonstrated by a large number of studies [37,38]. Crizotinib targets the receptor MET, which is frequently activated in human cancers promoting tumor growth, invasion and dissemination [39]. Sorafenib inhibits RAF, a protein-serine/threonine kinases that participate in the RAS-RAF-MEK-ERK signal transduction cascade [40]. Activation of MET receptor and RAF triggers several cellular processes such as cell survival, motility, adhesion, and apoptosis [39,40]. In our plant model, the ability of Crizotinib to specifically inhibit GFPChi sorting at low doses indicates target specificity. Moreover, the effects observed on microtubules may support the hypothesis that Crizotinib displays a more general cytotoxic activity. Sorafenib seems to affect MT polymerization only in some cases, possibly dependent on the specific physiological condition. Its effect on export from the ER (lysosomal targeting) is more generalized. The effect of both drugs on the endomembrane system is particularly interesting. The transport of proteins between the different compartments of the endomembrane system functions in plants as those in other eukaryotes [41]. Main components of this system are the ER, the Golgi apparatus, trans-Golgi network (TGN), peroxisomes, lytic compartments (vacuoles or lysosomes) and endosomes. This complex and dynamic intracellular network is a central player in many human cellular processes, including apoptosis and autophagy [42]. In particular, ER stress, Golgi and lysosome trafficking have been reported to be involved in the formation of autophagosomes [43].

In *A. thaliana*, Crizotinib and Sorafenib showed specific effects on trafficking pathways. Crizotinib in particular affected AleuGFP accumulation inducing a distribution pattern reminiscent of autophagocytosis events [44] while redirecting GFPChi from ER-to-vacuole direct transport to the apoplast. This fact is in accordance with previous experimental findings [45,46].

A significant potential issue when developing novel drug combinations is the unexpected increase in side effects due to overlapping toxicities or unpredictable drug-drug interactions. Interestingly, our analyses indicated a possible synergy between drugs. Crizotinib and Taxol showed synergistic effects on MTs organization; these effects should depend from the interaction on the same target or other possible target-related networks. In human cells, Crizotinib significantly enhanced the cytotoxicity of Taxol by reversing the *P*-glycoprotein mediated efflux of the drug [47]. In *A. thaliana*, 22 ABCB/multidrug resistance/pglycoprotein (MDR or PGP) were identified [48]. As mammalian PGP orthologs, this protein family is associated with multiple drug resistance, and localized to the plasma membrane where it is thought to function as detoxifying efflux pumps [48]. This opens the possibility to use plant for the screening of *P*-glycoprotein pump molecules, which would selectively inhibit the activity of that transporter.

The effects of other drug combinations have not yet been described in the literature and require more experiments to investigate their combined effects on membrane trafficking.

In conclusion, we demonstrated that *A. thaliana* is sensitive to a set of clinically relevant drugs including cytotoxic drugs and kinase inhibitors. Taken together, our results demonstrate the promising utility of this approach for identifying new potential anticancer drugs to be further characterized on specific human targets. This approach should be considered in the future for a cost-effective systemic study of other cytotoxic or molecular targeting agents.

## 4. Experimental Section

### 4.1. Chemicals

Crizotinib was purchased from Selleckchem (Houston, TX, USA), Taxol and Y-27632 from Tocris (Bristol, UK), Sorafenib was kindly provided by Pasquale Simeone. Crizotinib, Taxol, and Sorafenib were dissolved in dimethylsulfoxide (DMSO), Y-27632 in water.

### 4.2. Transgenic Plants and Confocal Microscopy

Chimerical GFP-tagged markers were stably expressed in *A. thaliana*, as previously described. GFP-tagged α-tubulin TUA6 (GFP-TUA6) was expressed in transgenic Arabidopsis ecotype Columbia under the control of the CaMV 35S promoter [22]. Three soluble GFPs (GFPKDEL; AleuGFP; GFPChi) differently sorted in the endomembrane system were expressed in transgenic *Arabidopsis* (cv. Wassilewskaja) under the control of the CaMV 35S promoter [23].

Transgenic plantlets were grown from T2 seeds on sterile solid Murashige and Skoog basal medium (MS, 3% sucrose, 0.8% agar) under continuous light (about 120 uE m-2 sec-1) at 24 °C. Observed samples consisted of plantlets transferred to liquid medium, supplemented with drugs in a range of concentration that is normally used to treat human cancer cells *in vitro*, into multiwell plates 14 days after germination and monitored in the following 24–36 h.

Full plantlets or primary leaves including petiole were mounted for fluorescence microscopical observation in water under glass coverslips and imaged using a confocal laser-microscope LSM 710 (Carl Zeiss MicroImaging GmbH, Germany). GFP markers were detected in the wavelength range 505–530 nm, assigning the green color, chlorophyll autofluorescence was detected above 650 nm, assigning the blue color. Excitation wavelength of 488 was used.
